# Outcomes of management of primary benign aggressive or malignant bone tumors around the elbow by limb-salvage surgery

**DOI:** 10.1186/s40634-023-00675-z

**Published:** 2023-10-23

**Authors:** Walid Atef Ebeid, Ismail Tawfeek Badr, Mohamed Kamal Mesregah, Bahaa Zakarya Hasan

**Affiliations:** 1 Department of Orthopaedic Surgery, Faculty of Medicine, Cairo University, Cairo, Egypt; 2 Department of Orthopaedic Surgery, Faculty of Medicine, Menoufia University, Shebin El-Kom, Menoufia, Egypt

**Keywords:** Limb salvage, Elbow tumors, Distal humerus tumors, Benign aggressive tumors, Malignant bone tumors, MSTS score

## Abstract

**Purpose:**

Bone tumors around the elbow are rare, with frequently delayed diagnosis. The current study aimed to assess the functional and oncological outcomes of limb salvage surgery for primary benign aggressive or malignant bone tumors around the elbow.

**Methods:**

We conducted a retrospective review of patients with primary aggressive benign and malignant bone tumors around the elbow treated with limb salvage surgery between 1995 and 2020 at a single musculoskeletal oncology center. The minimum follow-up period was 24 months. Functional results were assessed using the Musculoskeletal Tumor Society (MSTS) scoring system at the last follow‐up visit. Local recurrence, chest metastasis, and complications were recorded.

**Results:**

This study included 30 patients, 19 males and 11 females, with a mean age of 25.4 ± 14.2 years. The tumor location was the distal humerus (*n* = 21), proximal radius (*n* = 5), and proximal ulna (*n* = 4). Reconstruction was done by elbow fusion using fibular graft (*n* = 10), mobile endoprosthesis (*n* = 9), excision arthroplasty (*n* = 7), and extracorporeal freezing and reimplantation (*n* = 4). The mean follow-up period was 36.2 ± 21.3 months. The median follow-up MSTS score was 27 [Interquartile range (IQR): 26–30]. Skeletally immature patients had a significantly higher MSTS score. The rate of postoperative complications was 26.7%.

**Conclusion:**

Limb salvage surgery with different reconstructive options for benign aggressive and malignant bone tumors around the elbow can achieve good functional and oncological outcomes.

**Level of evidence:**

Level IV.

## Introduction

Bone tumors around the elbow are a rare entity, with an incidence of about 1% of all osseous tumors [27]. The most frequent malignant tumors of the elbow are Ewing sarcoma, osteosarcoma, and chondrosarcoma, most commonly affecting the distal humerus in older patients [4].

For a proper diagnosis, a detailed history, thorough physical examination, and radiological imaging are crucial. Symptoms of malignancy typically include unexplained unremitting rest pain, swelling, or fracture [6, 7, 13]. Diagnostic imaging modalities include plain X-rays, CT, and MRI, in addition to chest CT and bone scan for detection of distant metastasis [9]. Moreover, core-needle or open biopsy with histopathological specimen should be obtained to confirm the diagnosis before proceeding with definitive treatment [25, 30].

Due to the limited soft tissue envelope and proximity of neurovascular structures, treating primary benign aggressive or malignant bone tumors around the elbow can be more challenging than in other body areas. As a result of these anatomic considerations, amputation was traditionally the treatment of choice [27].

Advancement in adjuvant chemotherapy combined with en bloc tumor resection have improved the management and prognosis of patients with malignant tumors, such as Ewing sarcoma and osteosarcoma [2, 3, 11].

Several limb salvage and reconstruction procedures are available, including autografts, allografts, megaprostheses, allograft-prosthetic composites, and arthrodesis [5, 13, 22]. Surgical treatment requires careful preoperative planning and consideration of various possible resection and reconstruction techniques based on tumor size and location [5].

Elbow reconstruction is demanding, and the options for reconstruction can be limited given that the elbow joint is a complex interaction between several joints that must be stabilized for optimal wrist and hand function. In addition, achieving safe oncological margins can be challenging [13].

The existing literature about primary bone tumors of the elbow is quite sparse [4, 15]. The current study sought to evaluate the functional and oncological results of limb salvage surgery for primary benign aggressive or malignant bone tumors around the elbow.

## Methods

This study was a retrospective review of clinical, histopathological and radiological records of patients with primary aggressive benign or malignant bone tumors around the elbow, that were managed by limb salvage surgery, operated on between 1995 and 2020 at a single musculoskeletal oncology center. Approval from the Institutional Review Board (IRB) was obtained prior to the conduction of the study. All methods were performed in accordance with the relevant guidelines and regulations.

Patients with tumors around the elbow that were not amenable to limb salvage and were thus treated by above-elbow amputations or shoulder disarticulations were excluded from the study. Patients with soft tissue tumors were also excluded. The minimum follow-up period was 24 months.

Collected preoperative data included age, sex, the bone affected, the histopathological diagnosis, previous surgical interventions, the presence of pathological fracture, and the findings of radiological investigations, including plain X-rays, CT, MRI, bone scan, and chest CT.

Operative data included the type and length of resection, the operative margins, the type of limb salvage surgery, reconstruction technique, and operative time. Tumor location, size, soft tissue extent, skin condition, and relationship to the neurovascular bundle determined the type of limb-salvage surgery performed.

During follow-up visits, clinical evaluation for oncological and functional outcomes was done. Chest CT and bone scan were obtained for detection of distant metastasis. Plain X-rays were done to assess the radiologic outcome of the reconstruction method as well as possible detection of local recurrence. MRI and CT were required when a local tumor recurrence was suspected.

Functional outcomes were evaluated using the Musculoskeletal Tumor Society (MSTS) scoring system [8] at the last follow‐up visit. The recurrence rate, chest metastasis, and complications were reported.

### Statistical analysis

Statistical analysis was performed using IBM SPSS version 26.0 (Armonk, NY: IBM Corp). Qualitative variables were reported as frequency and percentage. Continuous variables were reported as mean and standard deviation (SD), or median and interquartile range (IQR), when appropriate. The Mann–Whitney U test or Kruskal–Wallis tests were used to compare the MSTS scores between groups when appropriate. The significance level was set at *p*-values less than 0.05.

## Results

### Epidemiological and baseline characteristics

This study included 30 patients, 19 (63.3%) males and 11 (36.7%) females, with a mean age of 25.4 ± 14.2 (range, 4–56) years. Overall, 24 (80%) patients were skeletally mature, and 6 (20%) patients were skeletally immature.

The tumor was located in the distal humerus (*n* = 21, 70%), proximal radius (*n* = 5, 16.7%), and proximal ulna (*n* = 4, 13.3%). Twenty-one (70%) patients had de novo tumors, while 9 (30%) patients presented as recurrent cases after previous surgery at other hospitals.

The histopathological diagnosis was Ewing sarcoma (*n* = 8), osteosarcoma (*n* = 5), chondrosarcoma (*n* = 4), synovial sarcoma (*n* = 4), giant cell tumor (*n* = 3), angiosarcoma (*n* = 2), epithelioid sarcoma (*n* = 1), parosteal osteosarcoma (*n* = 1), osteoblastoma (*n* = 1), and aneurysmal bone cyst (*n* = 1). Two (6.7%) patients presented with pathological fractures, Table 1.

**Table 1:** Baseline and demographic data of the included patients

Characteristics	Study patients (*n* = 30)
**Age**, years (mean ± SD)	25.4 ± 14.2
**Gender** (n, %)	
Males	19 (63.3%)
Females	11 (36.7%)
**Skeletal maturity** (n, %)	
Mature	24 (80%)
Immature	6 (20%)
**Tumor location** (n, %)	
Distal humerus	21 (70%)
Proximal radius	5 (16.7%)
Proximal ulna	4 (13.3%)
**Recurrent at presentation** (n, %)	
De novo	21 (70%)
Recurrent	9 (30%)
**Histopathological diagnosis** (n, %)	
Ewing sarcoma	8 (26.7%)
Osteosarcoma	5 (16.7%)
Chondrosarcoma	4 (13.3%)
Synovial sarcoma	4 (13.3%)
Giant cell tumor	3 (10%)
Angiosarcoma	2 (6.7%)
Epithelioid sarcoma	1 (3.3%)
Parosteal osteosarcoma	1 (3.3%)
Osteoblastoma	1 (3.3%)
Aneurysmal bone cyst	1 (3.3%)
**Pathological fractures** (n, %)	
No	28 (93.3%)
Yes	2 (6.7%)

### Operative data

The mean length of resection was 13.8 ± 5.6 (range, 8–34) cm. Intraarticular resection was done in 25 (83.3%) patients, extraarticular resection in 3 (10%) patients and hemicortical intercalary resection in 2 (6.7%) patients. Wide margin was achieved in all patients except 6 patients who had marginal margin in some parts.

**Fig. 1:**
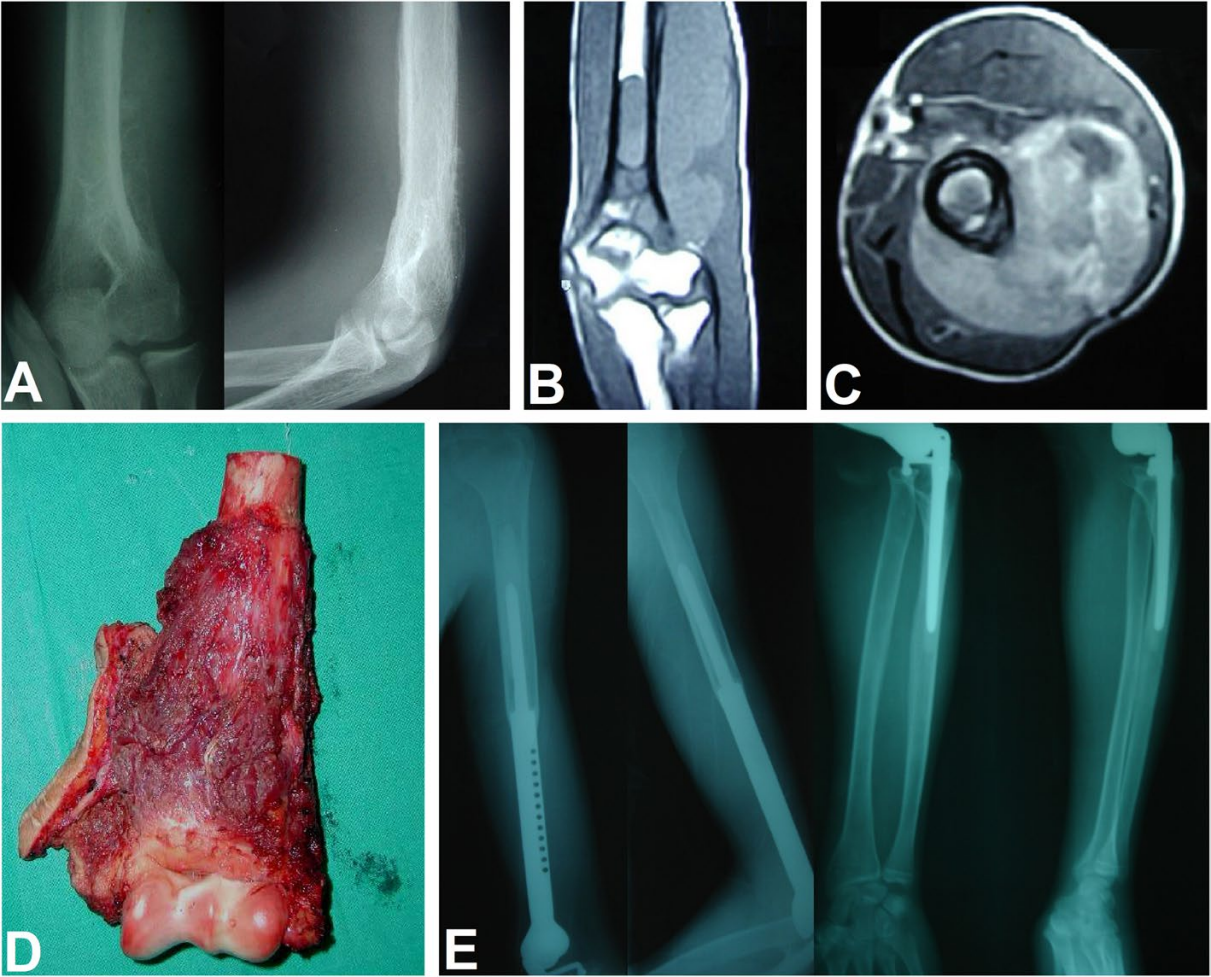
A 19-year-old male patient with Ewing's sarcoma of the distal humerus treated with wide resection and reconstruction by modular endoprosthesis. **A** Preoperative X-rays anteroposterior and lateral views. **B** Preoperative MRI coronal view. **C** Preoperative MRI axial view. **D** Intraoperative photograph of the resected distal humerus. **E** Six-year follow-up X-rays anteroposterior and lateral views

**Fig. 2:**
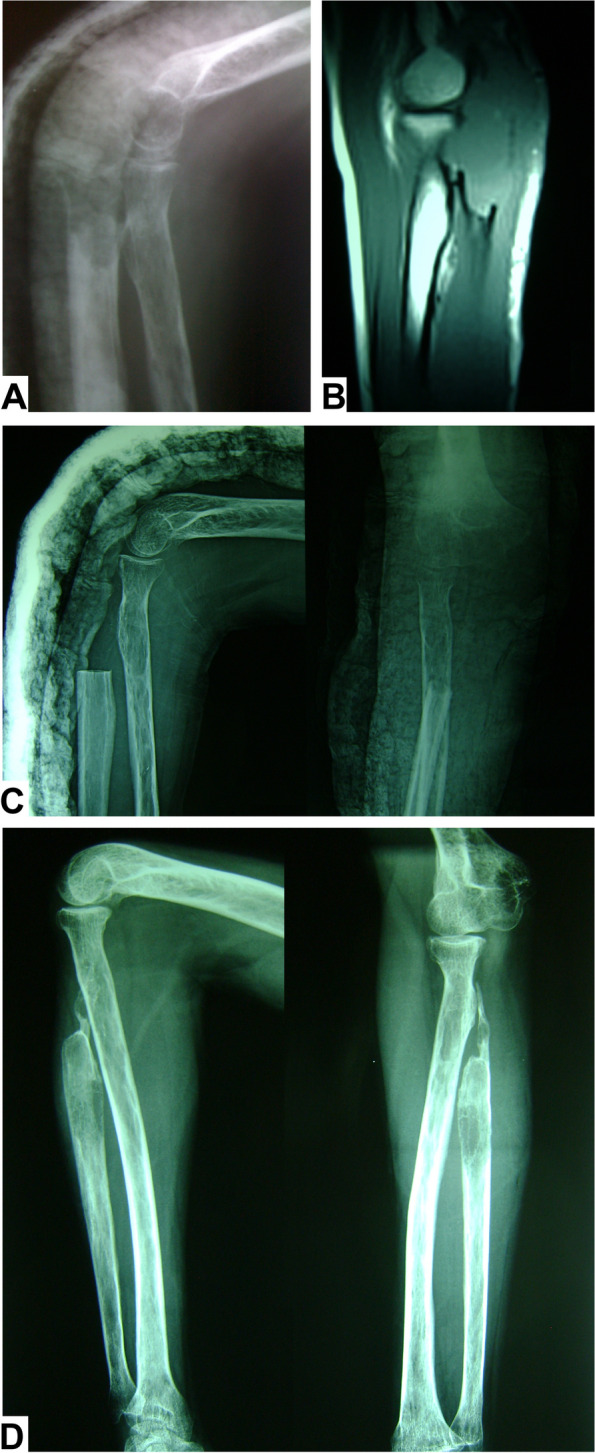
A 20-year-old male with Ewing's sarcoma of the proximal ulna treated by excision arthroplasty. **A** Preoperative X-ray lateral view. **B** Preoperative MRI sagittal view. **C** Immediate postoperative X-rays anteroposterior and lateral views. **D** Three-year follow-up X-rays anteroposterior and lateral views

**Fig. 3:**
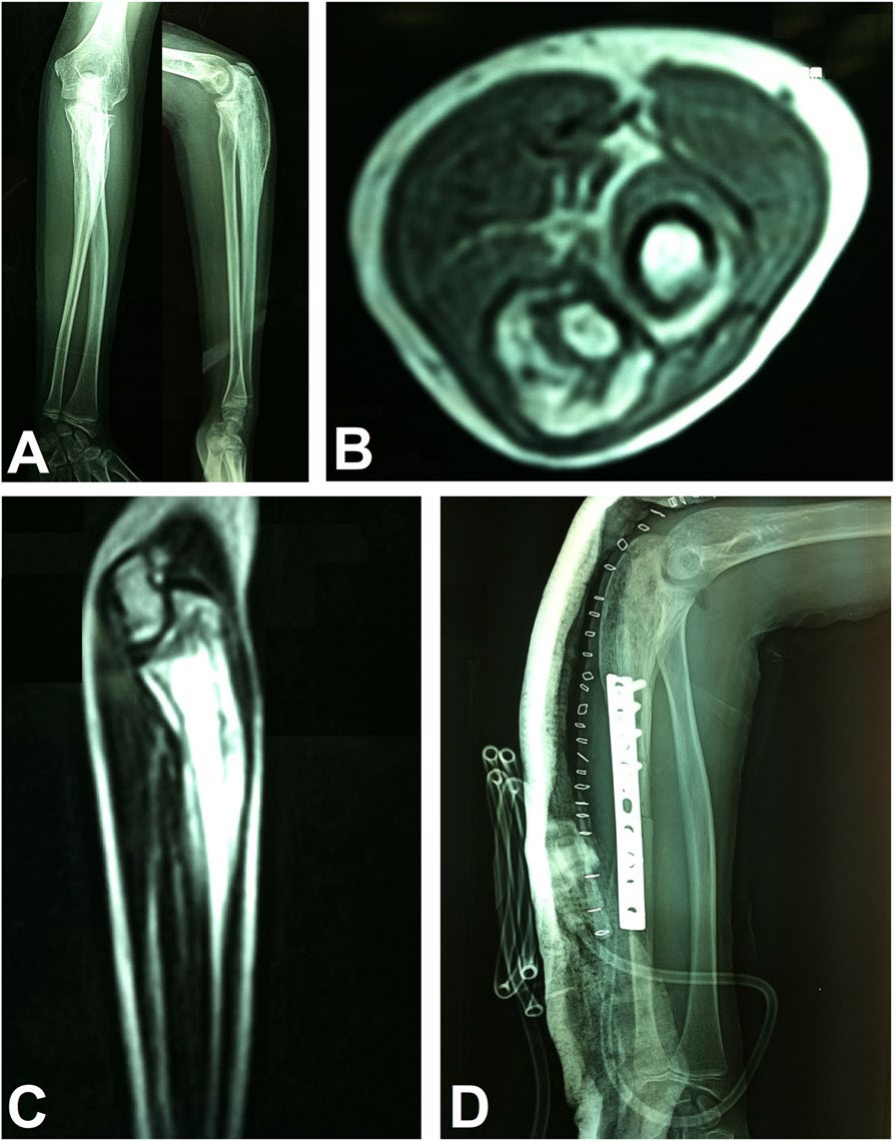
A 12-year-old female with Ewing's sarcoma of the proximal ulna treated by resection, extracorporeal freezing and reimplantation with plate fixation. **A** Preoperative X-rays anteroposterior and lateral views. **B** Preoperative MRI axial view. **C** Preoperative MRI sagittal view. **D** Postoperative X-ray lateral view

After wide resection, elbow fusion using vascularized or non-vascularized fibular graft was done in 10 (33.3%) patients, mobile endoprosthetic reconstruction in 9 (30%) patients, Fig. 1, excision arthroplasty in 7 (23.3%) patients, Fig. 2, and extracorporeal freezing, reimplantation and plate osteosynthesis in 4 (13.3%) patients, Fig. 3. The mean operative time was 4.6 ± 2.5 (range, 2–10) hours.

### Functional and oncologic outcomes

The mean follow-up period was 36.2 ± 21.3 (range, 24–105) months. The median last follow-up MSTS score was 27 (IQR: 26–30) points. Skeletally immature patients at presentation had a significantly higher median MSTS score compared to skeletally mature patients, 30 (IQR: 30–30) and 26 (IQR: 26–28.5), respectively, *P*<0.001. Other factors, including gender, history of previous surgery, tumor location, and method of reconstruction did not affect the MSTS score, Table 2.

**Table 2:** Factors affecting the functional outcomes

**Variables**	**MSTS score** Median (IQR)	***P*-value**
**Skeletal maturity**		**<0.001**
Mature (*n* = 24)	26 (26–28.5)	
Immature (*n* = 6)	30 (30–30)	
**Gender**		0.119
Males (*n* = 19)	26 (26–29)	
Females (*n* = 11)	29 (26–30)	
**Previous surgery**		0.349
De novo (*n* = 20)	27 (26–29.5)	
Recurrent (*n* = 10)	28 (26–30)	
**Tumor location**		0.815
Distal humerus (*n* = 21)	27 (26–30)	
Proximal radius or ulna (*n* = 9)	26 (26–30)	
**Method of reconstruction**		0.089
Elbow fusion (*n* = 10)	29 (26–30)	
Endoprothestic reconstruction (*n* = 9)	26 (25.5–27)	
Excision arthroplasty (*n* = 7)	26 (26–30)	
Extracorporeal freezing (*n* = 4)	29 (27.5–30)	

Postoperative complications occurred in 8 (26.7%) patients. Six patients had postoperative infection. One patient with Ewing sarcoma of the proximal ulna who had extracorporeal freezing had early wound infection and skin sloughing that responded to serial debridement and irrigation and excision of the proximal ulna to allow wound closure. Three patients with tumors of the distal humerus who had elbow fusion with fibular graft developed infection; one responded to antibiotics and serial debridement, the second improved after debridement and plate removal, and the third patient had septic nonunion which responded to repeated debridement and was kept in a brace.

Two patients with tumors of the distal humerus who had mobile endoprosthetic reconstruction developed infection; one had septic loosening of the prosthesis and was managed by prosthesis removal and cement spacer insertion, and the other patient responded to serial debridement without prosthesis removal.

One patient with osteosarcoma of the proximal radius who had wide resection developed wrist drop, which was managed by tendon transfer. Another patient with osteosarcoma of the proximal radius who had had elbow fusion with non-vascularized fibula developed distal nonunion of the fibular graft, and iliac crest bone grafting was done.

At the end of the follow-up period, only two (6.7%) patients with distal humerus tumors who had elbow fusion by vascularized fibula developed local recurrence and were managed by shoulder disarticulation. Three (10%) patients had chest metastasis; one died of disease, and the other two survived free of disease after lung metastasectomy.

## Discussion

Management of malignant tumors around the elbow is challenging, and there are several factors to consider when deciding between amputation and limb-salvage resection, such as the tumor site, size, extramedullary extension, distant metastasis, and the patient's characteristics. In terms of reconstruction, the options are limited and technically demanding due to the complex anatomy and biomechanics of the elbow, in addition to the proximity to the neurovascular bundles and limited soft tissue envelope [13, 15]. Various reconstruction options include fibular autografts, allografts, endoprosthetic replacements, total elbow arthroplasty, allograft-prosthesis composite, or arthrodesis [13, 24, 28, 31].

This study evaluated the functional and oncological outcomes of treating primary benign aggressive or malignant bone tumors around the elbow by limb salvage surgery. The median MSTS score improved significantly at the last follow-up, with a higher median score in skeletally immature patients. Overall, 26.7% of patients had complications, 6.7% had local recurrences, and 10% had chest metastases.

In our study, the most common tumor location was the distal humerus. Ewing sarcoma and osteosarcoma were the most common tumors. In Halai et al. [15] series of primary osseous tumors of the elbow, the distal humerus was the most common location and high-grade osteosarcoma and Ewing's sarcoma accounted for 61% and 25% of malignancies, respectively.

In our study, the functional outcomes were satisfactory, with a median MSTS score of 27 after a mean follow-up period of 36.2 months. Patients with biological reconstruction and elbow arthrodesis and those with mobile reconstructions using endoprosthesis had satisfactory and almost similar functional outcomes. The functional outcome was better in skeletally immature patients. This may be attributed to their good potential for healing, as 5 out of 6 skeletally immature patients received biological reconstruction.

Tang et al. [29] evaluated the outcomes of custom-made endoprosthetic reconstruction after resection of elbow tumors and reported an average MSTS score of 23.9. Similarly, Hanna et al. [16] reported a mean MSTS score of 22.7 after treating 18 patients with distal humerus tumors with endoprosthetic replacement. Kulkarni et al. [23] reported satisfactory outcomes following endoprosthetic reconstruction for tumors of the distal humerus. Henrichs et al. [17] reported a mean MSTS score of 24 after distal humeral endoprosthetic reconstruction.

Athwal et al. [1] treated 20 patients who had elbow tumors with total elbow arthroplasty and reported good pain relief and functional outcomes.

Kimura et al. [21] reported excellent elbow function four years after treating Ewing sarcoma of the proximal ulna in an eight-year-old girl with wide excision and vascularized fibular graft reconstruction. Kalaiah et al. [18] treated a 30-year-old male with giant cell tumor of the proximal ulna with a free fibular graft and reported satisfactory outcomes and stable functional joint two years after surgery. Graci et al. [12] reported excellent functional outcomes ten years following treatment of parosteal osteosarcoma of the distal humerus in a 12-year-old girl by en bloc resection, extracorporeal irradiation and reimplantation.

However, different reconstruction options have their own drawbacks. There is a risk of implant failure and loosening with total elbow arthroplasty in patients with extensive defects [22]. Endoprosthetic replacement cannot provide good elbow function compared to total elbow arthroplasty [23]. In allograft elbow reconstructions, the outcome is unpredictable, and complications are common such as graft nonunion [19, 20, 26]. Fibular autografting has the disadvantage of donor site morbidity [10]. Extracorporeal irradiation and reimplantation can be associated with infection and long-term arthritis [14].

In our study, the rate of local recurrence was 6.7% and the rate of chest metastasis was 10%. These rates were lower than the rates reported in Halai et al. [15] series, in which the rate of local recurrence of malignant tumors was 39%, and the rate of distant metastases was 43%.

In our study, complications occurred in 26.7% of patients. The main complication was infection, as many of these patients were immunocompromised and had extensive surgeries. However, the complications were manageable without affecting limb survivorship. Kruckeberg et al. [22] reported a 45% rate of complications after treating tumors of the distal humerus with resection and total elbow arthroplasty. Henrichs et al. [17] reported a 55% complication rate after endoprosthetic reconstruction.

We believe that limb salvage surgery for upper limb tumors, when performed with adequate resection margins, will offer a superior functional outcome than amputation without jeopardizing the oncologic outcome.

This study is not without limitations, including the retrospective nature and the absence of a control group. Moreover, due to the rarity of tumors around the elbow, we had to include various pathologies, locations, presentations and limb salvage techniques. Additionally, patients were enrolled in this study over a long period of time with changes in treatment methods, techniques, technology and recommendations over the years which could have affected the outcomes.

## Conclusion

Limb salvage surgery for benign aggressive and malignant bone tumors around the elbow is feasible with good functional and oncological outcomes. The different reconstructive options yield similar functional outcomes, with younger patients doing better. Most elbow bone tumors are manageable without jeopardizing limb survivorship.

## Data Availability

The dataset analyzed in this study is available from the corresponding author on request.
